# Development of engineered IL-36γ-hypersecreting *Lactococcus lactis* to improve the intestinal environment

**DOI:** 10.1007/s11274-024-04157-x

**Published:** 2024-10-24

**Authors:** Masahiro Yoda, Shogo Takase, Kaho Suzuki, Aito Murakami, Fu Namai, Takashi Sato, Tadashi Fujii, Takumi Tochio, Takeshi Shimosato

**Affiliations:** 1 Department of Biomolecular Innovation, Institute for Biomedical Sciences, Shinshu University, Nagano, 399-4598 Japan; 2 Food and Feed Immunology Group, Laboratory of Animal Food Function, Graduate School of Agricultural Science, Tohoku University, Sendai, Miyagi 980-8572 Japan; 3 Department of Medical Research on Prebiotics and Probiotics, Fujita Health University, Toyoake, Aichi 470-1101 Japan; 4 Institute for Aqua Regeneration, Shinshu University, Nagano, 399-4598 Japan; 5 Department of Pharmacy, Medical Faculty, Universitas Brawijaya, Malang, 65145 Indonesia

**Keywords:** gmLAB, IL-36γ, Next-generation sequencing, Gut microbiota, *Muc2*

## Abstract

Interleukin (IL) 36 is a member of the IL-1-like proinflammatory cytokine family that has a protective role in mucosal immunity. We hypothesized that mucosal delivery of IL-36γ to the intestine would be a very effective way to prevent intestinal diseases. Here, we genetically engineered a lactic acid bacterium, *Lactococcus lactis*, to produce recombinant mouse IL-36γ (rmIL-36γ). Western blotting and enzyme-linked immunosorbent assay results showed that the engineered strain (NZ-IL36γ) produced and hypersecreted the designed rmIL-36γ in the presence of nisin, which induces the expression of the recombinant gene. We administered NZ-IL36γ to mice via oral gavage, and collected the ruminal contents and rectal tissues. Colony PCR using primers specific for NZ-IL36γ, and enzyme-linked immunosorbent assay to measure the rmIL-36γ concentrations of the ruminal contents showed that NZ-IL36γ colonized the mouse intestines and secreted rmIL-36γ. A microbiota analysis revealed increased abundances of bacteria of the genera *Acetatifactor*, *Eubacterium*, *Monoglobus*, and *Roseburia* in the mouse intestines. Real-time quantitative PCR of the whole colon showed increased *Muc2* expression. An in vitro assay using murine colorectal epithelial cells and human colonic cells showed that purified rmIL-36γ promoted *Muc2* gene expression. Taken together, these data suggest that NZ-IL36γ may be an effective and attractive tool for delivering rmIL-36γ to improve the intestinal environment.

## Introduction

Gut mucosal barriers separate the external environment from the internal milieu of the body (Okumura and Takeda 2018). These selectively permeable barriers prohibit the passage of bacteria and toxins while permitting the flux of water, ions, and solutes, including nutrients (France and Turner 2017). The complex homeostasis of gut mucosal barriers is maintained by the host immune response and gut microbiota (Morrison and Preston 2016; Chen et al. 2021; Ngo et al. 2021). Interestingly, intestinal homeostasis is regulated by proinflammatory cytokines, such as interleukin (IL) 36, which is part of the IL-1 superfamily (Bassoy et al. 2018). There are three members of the IL-36 family, i.e., IL-36α, IL-36β, and IL-36γ, which act as IL-36 receptor agonists and activate or regulate inflammatory pathways, including nuclear factor kappa B (NF-κB) (Bassoy et al. 2018; Murrieta-Coxca et al. 2019). It has been reported that the IL-36 family increases *Muc2* expression, suggesting that IL-36 cytokines can act directly to increase mucus production (Giannoudaki et al. 2019). Other studies have indicated that IL-36γ functions as a global epithelial alarmin and broad sensor of pathogenic infection (Macleod et al. 2020), and that induced IL-36γ influences neutrophil accumulation, cytokine production, and the repair of intestinal damage following injury (Medina-Contreras et al. 2016; Ngo et al. 2018). IL-36 cytokines have been shown to play a role in not only the regulation of inflammatory pathways, but also in changes in the microbiota composition (Giannoudaki et al. 2019).

Through recent advances in next-generation sequencing-based meta-genomics, it has been found that host genetics play a critical role in shaping the diverse microbial communities in the gut, and the microbial communities have been implicated in intestinal homeostasis maintenance (Gu et al. 2019; Martin-Gallausiaux et al. 2021). For example, *Acetatifactor* and *Eubacterium* have been found to affect host mucosal barriers by regulating several inflammatory pathways, including NF-κB (Bassoy et al. 2018; Murrieta-Coxca et al. 2019). *Monoglobus* species have the ability to ferment dietary fiber to produce butyrate, which inhibits inflammation and oxidative stress. In addition, *Roseburia* species have been associated with intestinal protection from inflammatory diseases, such as ulcerative colitis (UC) (Ordás et al. 2012; Vermeiren et al. 2012; Machiels et al. 2017) and Crohn’s disease (CD) (Willing et al. 2010; Chen et al. 2014; Petagna et al. 2020). Thus, these kinds of bacteria play key roles in maintaining intestinal homeostasis.

Lactic acid bacteria are widely used in industry food fermentation, and they contribute to the flavor and preservation of fermented products (Samazan et al. 2015). Advances in cell-engineering technology have extended the potential of the use of genetically modified strains of lactic acid bacteria (gmLAB) (Morello et al. 2008; Samazan et al. 2015). Such strains are used to produce various biomedical proteins (Shigemori et al. 2017; Namai et al. 2020a; Oshima et al. 2024), biological mediators (hormones and ILs) (Steidler et al. 2003; Namai et al. 2018), and molecules that stimulate immune responses (Huibregtse et al. 2007; Ai et al. 2014; Ren et al. 2014). Thus, we hypothesized that the mucosal delivery of gmLAB engineered to produce IL-36γ in the intestine might be an effective method for maintaining intestinal homeostasis and preventing intestinal diseases.

In the present study, we constructed a gmLAB that secretes recombinant mouse IL-36γ (rmIL-36γ) by introducing a gene expression vector into *Lactococcus lactis*, and we verified the viability of the gmLAB in the mouse digestive tract and rmIL-36γ secretion from the gmLAB in C57BL/6 mice. We also investigated the changes in the intestinal microbiome and *Muc2* expression induced by gmLAB administration. In addition, the bioactivity of the generated rmIL-36γ was verified by assaying for *Muc2* expression in murine colorectal epithelial and human colonic cell lines. Our results indicated that the oral administration of rmIL-36γ-secreting gmLAB might be a very effective way to improve the intestinal environment.

## Materials and methods

### Bacteria, growth conditions, and plasmid

**Fig. 1:**
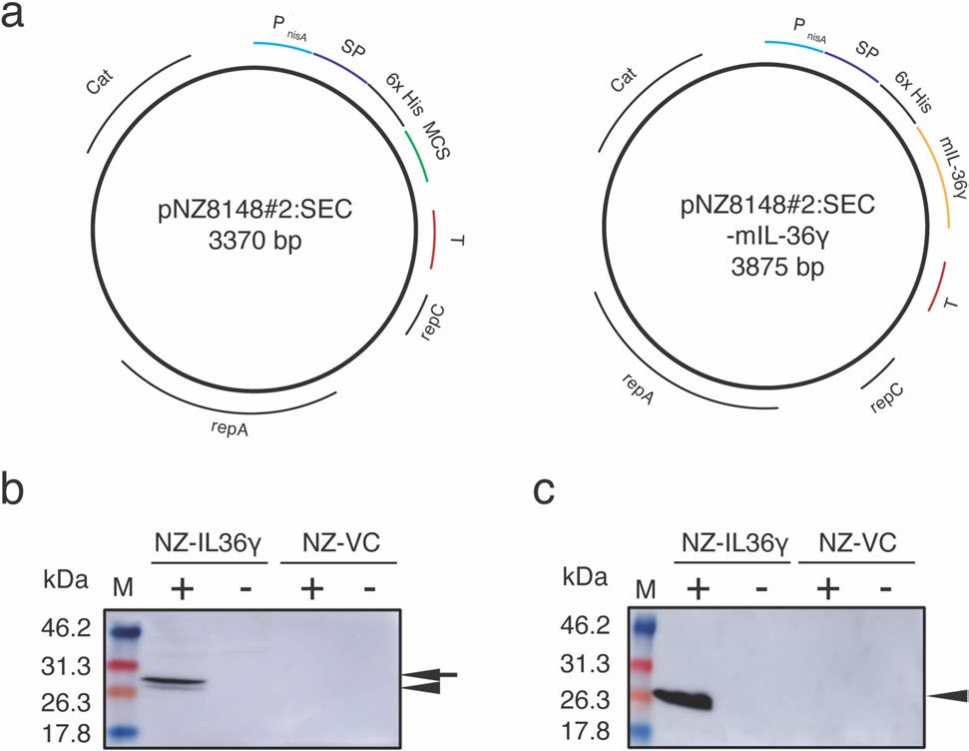
The construction of gmLAB and the recombinant gene expression analysis. **a** The lactococcal gene expression vector, pNZ8148#2:SEC (left). *P*_*nisA*_: nisin-inducible promoter, *SP*: signal peptide, *6x His*: 6x histidine tag, *MCS*: multi-cloning site, *T*: terminator, *repC and repA*: the origin of replication, *Cat*: chloramphenicol acetyltransferase. The mIL-36γ expression vector, pNZ8148#2:SEC-mIL-36γ (right). The human *mIL-36γ* gene was inserted into the MCS of pNZ8148#2:SEC. mIL-36γ: the sequence encoding mouse IL-36γ. **b**,** c** The cellular extract (b) and culture supernatant (c) samples from gmLABs (NZ-IL36γ and NZ-VC) were analyzed by western blotting using an anti-His-tag Ab. The arrowhead indicates the signal peptide-conjugated form of rmIL-36γ (27.8 kDa). The arrow indicates the signal peptide-cleaved form of rmIL-36γ (25.0 kDa). Gene expression was induced in NZ-IL36γ and NZ-VC with (+) or without (-) nisin. M: molecular mass markers (kDa)

We used *L. lactis* subsp. *cremoris* NZ9000 (NZ9000; MobiTec, Goettingen, Germany) as the host for recombinant protein expression. NZ9000, which was derived from *L*. *lactis* subsp. *cremoris* MG1363 by inserting genes encoding NisK and NisR into its genome, is suitable for use in the nisin-controlled gene expression (NICE) system (Mierau and Kleerebezem 2005). NZ9000 was cultured in M17 broth (BD Difco™, Becton, Dickinson and Company, Sparks, MD, USA) containing 0.5% glucose (GM17) at 30 °C without shaking. The constructed gmLAB cells were cultured in GM17 containing 10 µg/mL chloramphenicol (GM17cm). The NICE-system plasmid, pNZ8148#2SEC (Shigemori et al. 2014), was employed as the mouse IL-36γ (mIL-36γ, accession No: NM153511) gene expression vector, and was constructed from commercially available pNZ8148 (MoBiTec) (Shigemori et al. 2012). pNZ8148#2SEC harbors a nisin-inducible promoter, *P*_nisA_, an origin of replication for lactic acid bacteria, and a chloramphenicol resistance gene (Fig. 1a).

### Construction of a gmNZ9000 strain for mIL-36γ gene expression

General molecular cloning techniques were used according to previously described methods (Russell and Sambrook 2001). A DNA sequence encoding mIL-36γ was optimized for MG1363 codon usage and synthesized by Eurofins Genomics (Tokyo, Japan). The DNA fragment was amplified by polymerase chain reaction (PCR) using KOD-Plus-Neo DNA polymerase (TOYOBO, Osaka, Japan) and a primer pair (forward: 5’-TGACCTTCCGCAGATAAG-3’; reverse: 5’-CATCGTGAACATGCCAAC-3’) according to the manufacturer’s instructions. The resulting fragment was digested with the BamHI and HindIII restriction enzymes (Takara Bio, Shiga, Japan), then ligated with BamHI/HindIII-digested pNZ8148#2:SEC. The resulting plasmid (pNZ8148#2:SEC-mIL-36γ; DDBJ accession number LC823121) was analyzed by DNA sequencing (performed by Eurofins Genomics), and was consistent with the predicted sequence. pNZ8148#2:SEC-mIL-36γ was introduced into electrocompetent NZ9000 cells by electroporation using a Gene Pulser Xcell electroporation system (Bio-Rad, Hercules, CA, USA). The resulting gmNZ9000 strain was designated as NZ-IL36γ. NZ-VC (Shigemori et al. 2014), a derivative strain of NZ9000 harboring pNZ8148#2:SEC, was used as a vector control strain.

### Induction of recombinant gene expression in gmNZ9000 strains

The gmNZ9000 strains were cultured in 2-, 50-, or 1000-mL culture systems to induce recombinant gene expression. An overnight culture of gmNZ9000 was inoculated at a dilution of 1/20 into fresh GM17cm broth, and incubated until the turbidity reached an optical density at 600 nm (OD_600_) of around 0.4. Then, the culture was supplemented with or without nisin (MoBiTec) at a final concentration of 1.25 ng/mL, and further incubated for 3 h (2- and 50-mL scale cultures) or 24 h (1000-mL scale culture). To determine the level of rmIL-36γ at each time point, western blotting was performed as described below. The intensity of each band was measured using ImageJ (ImageJ version 1.51j8, NIH, Bethesda, MD, USA).

### Preparation of protein samples from the gmNZ9000 culture

After nisin induction, bacterial cells and culture supernatants were separated by centrifugation at 8000 × *g* and 4 °C for 5 min. Bacterial cells from 2-mL cultures were washed once with 1 mL of ice-cold Tris-buffered saline (TBS; 50 mM Tris, 138 mM sodium chloride, 2.7 mM potassium chloride, pH 8.0), then suspended in 400 µL of ice-cold TBS-containing protease inhibitor cocktail (Roche Diagnostics, Mannheim, Germany). The suspension was mixed with 0.4 g of glass beads (0.2 mm in diameter, AS ONE, Osaka, Japan), and shaken using a bead beater (lT-12, TAITEC, Saitama, Japan). The soluble fraction was collected by centrifugation at 20,400 × *g* and 4 °C for 15 min. Supernatant samples from the 2-mL cultures were prepared using trichloroacetic acid precipitation as described previously (Shigemori et al. 2014). An equal volume of 2× sample buffer solution (196-11022, Wako, Osaka, Japan) was added to some of the samples, and the mixtures were boiled for 5 min.

### Detection of rmIL-36γ

The protein samples were subjected to sodium dodecyl sulfate-polyacrylamide gel electrophoresis. The bands of separated proteins were visualized by gel staining with Coomassie brilliant blue (Polysciences, Warrington, PA, USA) or transferred from the gel onto a polyvinylidene fluoride membrane (GE Healthcare, Buckinghamshire, United Kingdom). The protein-blotted membrane was blocked with skim milk, reacted with mouse anti-His-tag antibody (Ab; 1/1000; 652501, BioLegend, San Diego, CA, USA), followed by incubation with horseradish peroxidase-conjugated goat anti-mouse IgG Ab (1/3000; A4416, Sigma-Aldrich, St. Louis, MO, USA). The resulting blots were reacted with ECL Prime Western Blotting Detection Reagent (GE Healthcare), and detected using a Lumino image analyzer (ImageQuant LAS 500, GE Healthcare).

### Quantitation of rmIL-36γ

The rmIL-36γ concentrations of the cellular extracts, supernatant samples, ruminal contents, and intestinal tissue samples were quantified using a Mouse IL36 gamma/IL-1F9 enzyme-linked immunosorbent assay (ELISA) kit (Abcam plc, Cambridge, United Kingdom) according to the manufacturer’s instructions. The dialyzed solution obtained following purification (as described below) was quantified using a His Tag ELISA Detection Kit (Funakoshi, Tokyo, Japan) according to the manufacturer’s instructions.

### Mice

Six-week-old C57BL/6 male mice were obtained from Japan SLC, Inc. (Hamamatsu, Japan) and housed under humidity- and temperature-controlled conditions with a 12-h light-dark cycle and *ad libitum* access to water and pellet food MF (Oriental Yeast Co., Tokyo, Japan).

### NZ-IL36γ viability and IL secretion in mouse intestines

After 1 week of acclimatization, 12 mice were randomly assigned to two groups: one group received 1 × 10^10^ colony-forming units (cfu)/200 µL of phosphate-buffered saline (PBS; 137 mM sodium chloride, 2.7 mM potassium chloride, 10 mM disodium hydrogen phosphate, and 1.76 mM potassium dihydrogen phosphate, pH 7.4) of NZ-IL36γ (NZ-IL36γ group; *n* = 6), and the other group was administered PBS (PBS group; *n* = 6) via oral gavage every 30 min for a total of 10 times in a day. At 30 min after the final (10th ) treatment, all mice were sacrificed under anesthesia. We collected the ruminal contents from the sigmoid colon (colon contents) and cecum (cecum contents), and tissue samples were collected from the rectal region approximately 1 cm before the anus (rectal tissues). The colon contents and cecum contents were each adjusted to 200 mg/mL (w/v) in PBS, and homogenized with a Beads Crusher µT-12 (lT-12, TAITEC) at 1600 rpm for 1 min. Then, 100 µL of each homogenized sample was diluted 100-fold in PBS, applied to GM17cm agar, and incubated at 30 °C for 48 h. Colony PCR (Bergkessel and Guthrie 2013) was performed using two primer pairs (Table 1) specific for pNZ8148#2:SEC-mIL-36γ and *L. lactis* subsp. *cremoris*. The generated PCR products were separated by size on a 1% agarose gel. For the measurement of the rmIL-36γ concentrations, the colon contents and cecum contents were homogenized, adjusted to 200 mg/mL (w/v) in PBS, and the supernatants were separated by centrifugation at 8000 × *g* and 4 °C for 5 min. The rectal tissues were also homogenized, and the supernatants were separated; the total protein concentrations of the supernatants were measured using a BCA Protein Assay kit (Thermo Fisher Scientific, Rockford, IL, USA) according to the manufacturer’s instructions, and were subsequently adjusted to 1500 mg/mL in PBS. The rmIL-36γ concentrations of the supernatant samples (colon contents, cecum contents, and rectal tissues) were quantified as described above.

**Table 1:** NZ-IL36γ-specific primer sequences

Name	5’-sequence-3’	Length
pNZ F	TGCCCCGTTAGTTGAAGAA	20
pNZ R	TCAATCAAAGCAACACGTGC	20
CreF	GTGCTTGCACCGATTTGAA	19
CreR	GGGATCATCTTTGAGTGAT	19

### Gut microbiota analysis

All feces were collected at the end of the administration period and used for the analyses of the microbiota (PBS group: *n* = 5; NZ-VC group: *n* = 5; and NZ-IL36γ group: *n* = 5). An aliquot of each fecal sample was mixed with the fecal collection kit for amplicon sequencing analysis by next-generation sequencing (TechnoSuruga Laboratory Co., Ltd., Tokyo, Japan). The 16 S V3-V4 region of the fecal DNA was amplified as described previously (Okajima et al. 2021). The constructed DNA library was sequenced using the MiSeq system (Illumina, Tokyo, Japan). Microbiota analysis was then performed using the Quantitative Insights into Microbial Ecology version 2 (Qiime2) platform (Bolyen et al. 2019). The resulting amplicon data were filtered, denoised, and merged using the divisive amplicon sequence variant table. Taxonomic annotation was performed using a classifier based on the Silva 138 database (Bokulich et al. 2018). The α-diversity was examined using Faith’s phylogenetic diversity (PD), observed amplicon sequence variants (observed features), and the Shannon diversity index. Principal coordinate analysis and significance difference testing using the β-diversity (Bray–Curtis) index were also performed. We used multivariate analysis by linear models 2 (MaAsLin2) (Mallick et al. 2021) to find associations between NZ-IL36γ administration and bacterial abundances. All *p* values were corrected for multiple testing using the false discovery rate (FDR) in MaAsLin2.

### Real-time quantitative RT-PCR (RT-qPCR)

Total RNA was isolated from the fecal samples and cells using Nucleospin RNA (MACHEREY-NAGEL, North Rhine-Westphalia, Germany). cDNA was synthesized using the PrimeScript^®^ RT reagent kit (Perfect Real Time, Takara Bio) according to the manufacturer’s instructions. RT-qPCR was performed with a Thermal Cycler Dice Real Time System (Takara Bio) and TB Green^®^ Premix Ex Taq™ II (Takara Bio). The reaction was performed in a final volume of 10 µL, which included 5 µL of 2× TB Green Premix Ex Taq II master mix containing primers specific for mouse or human *Muc2* and β-actin (Takara Bio; final concentration, 0.5 µM). The thermal cycle parameters included 45 cycles of amplification with 5 s at 95 °C for denaturation, and 30 s at 60 °C for annealing and elongation. Measurements were normalized using the β-actin (housekeeping gene) expression levels.

### **NZ-IL36γ administration and *Muc2 *RT-qPCR**

After 1 week of acclimatization, 15 mice were randomly assigned to three groups: one group was administered PBS (PBS group; *n* = 5), and the others were administered 1 × 10^10^ cfu/200 µL of PBS of NZ-IL36γ (NZ-IL36γ group; *n* = 5) or NZ-VC (NZ-VC group; *n* = 5) via oral gavage five times per week for 2 weeks (total of 10 times). Two weeks after the first dosing, all mice were sacrificed under anesthesia, and the whole colon was collected. RNA was extracted from the colon tissues in RNAlater solution (Invitrogen, Carlsbad, CA, USA). Colon tissues were homogenized using a Beads Crusher µT-12 (lT-12, TAITEC) at 2,500 rpm for 1 min. RNA extraction, cDNA synthesis, and qPCR were performed as described above.

### Purification of rmIL-36γ

Recombinant gene expression was induced by nisin in the 1000-mL cultures, as described above. The culture supernatant was collected by centrifugation at 8000 × *g* and 4 °C for 5 min, and an equal volume of 2× binding buffer (40 mM sodium phosphate and 1 M sodium chloride) was added. The pH was adjusted to 7.4 with sodium hydroxide, and the solution was passed through a membrane filter (pore size of 0.45 μm, Merck Millipore, Billerica, MA, USA). The filtrate was loaded onto a HisTrap HP column (1 mL, GE Healthcare) equilibrated with 1× binding buffer (20 mM sodium phosphate and 500 mM sodium chloride, pH 7.4). The column was washed with five times the column bed volume of 1× binding buffer. The proteins that had adsorbed on the column were then eluted with a linear gradient of 0 to 500 mM imidazole over 35 column bed volumes at 1 mL/min using a fast protein liquid chromatography system (AKTA pure 25, GE Healthcare). As described above, sodium dodecyl sulfate-polyacrylamide gel electrophoresis and western blotting were performed to analyze the collected fractions (culture supernatant, flow-through, wash, and eluate). The eluted fractions were dialyzed against PBS. The rmIL-36γ concentration in the concentrate was determined as described above.

### Culture conditions for CMT93/69 and HT-29 cells

Murine colorectal epithelial CMT93/69 cells (KAC Co., Ltd., Kyoto, Japan) (Franks and Hemmings 1978) and human colonic HT-29 cells (ATCC, Manassas, VA, USA) (Martínez-Maqueda et al. 2015) were cultured in 75-cm^2^ cell culture flasks (TPP, Trasadingen, Switzerland) in complete Dulbecco’s modified eagle medium (Thermo Fisher Scientific) containing 10% fetal bovine serum (GE Healthcare), 100 U/mL penicillin, and 100 µg/mL streptomycin (Nacalai Tesque, Kyoto, Japan) at 37 °C in a humidified incubator with 5% carbon dioxide; the cells were cultured until they reached 80–90% confluency on the growth surface in the flask.

### Bioactivity assay of purified rmIL-36γ

CMT93/69 cells and HT-29 cells were seeded on 24-well plates (Nippon Genetics, Tokyo, Japan) at a density of 2 × 10^5^ cells per well and cultured for 24 h. CMT93/69 cells were washed with PBS and stimulated for 3 h with 1 or 2 µg/mL of purified rmIL-36γ in PBS for the samples, or stimulated for 24 h with 1 or 2 µg/mL of rmIL-36γ and rmIL-1β (both from *Escherichia coli*, R&D Systems, Minneapolis, MN, USA) in PBS for the positive controls. HT-29 cells were stimulated for 24 h with 10 or 100 µg/mL of purified rmIL-36γ in PBS for the samples, or stimulated for 24 h with 10 or 100 µg/mL of rmIL-36γ or rmIL-1β (both from *E. coli*, R&D Systems) in PBS for the positive controls.

### Statistical analysis

Statistical analyses were performed using GraphPad Prism software (Version 7, GraphPad, San Diego, CA, USA). One-way analysis of variance and Tukey–Kramer tests were used to determine the significance of the differences. Differences were considered to be statistically significant at *p* < 0.05. Values are expressed as the means ± standard deviation (SD). Principal coordinate analysis and significance difference testing using the β-diversity index were also performed.

## Results

### Construction of rmIL-36γ-producing gmLAB

The mIL-36γ sequence was inserted into pNZ8148#2:SEC (Fig. 1a, left) using BamHI and HindIII digestion and ligation, and the resulting vector, pNZ8148#2:SEC-mIL-36γ (Fig. 1a, right), was sequenced to confirm that it did not contain any mutations or deletions (data not shown). pNZ8148#2:SEC-mIL-36γ was introduced into strain NZ9000 by electroporation to generate the mIL-36γ-producing gmLAB (designated as NZ-IL36γ cells). The original vector, pNZ8148#2:SEC, was also introduced into strain NZ9000 to generate the vector control gmLAB (designated as NZ-VC). Target gene expression by the gmLAB was induced with nisin, then western blotting using an anti-His tag Ab was performed.

Bands corresponding to the signal peptide-conjugated form of mIL-36γ (27.8 kDa) and the signal peptide-cleaved form of mIL-36γ (25.0 kDa) were detected in the cellular extract of NZ-IL36γ cells (Fig. 1b). In the supernatant of NZ-IL36γ cells, only the signal peptide-cleaved form was detected (Fig. 1c). In contrast, no band was detected in the NZ-VC samples, nor in the samples without nisin stimulation (Fig. 1b, c). These results indicated that NZ-IL36γ cells produced rmIL-36γ following nisin stimulation.

### Determination of the optimal culture time and protein concentration for rmIL-36γ expression

**Fig. 2:**
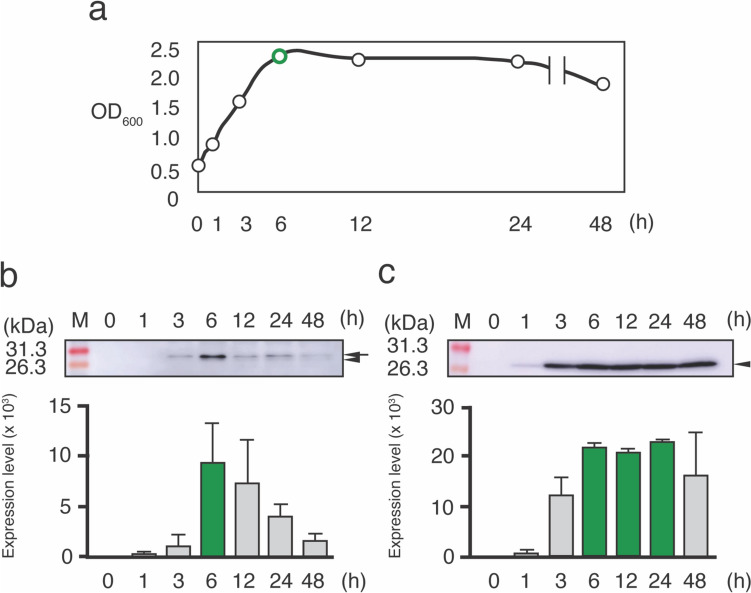
Analysis of the optimal culture time for rmIL-36γ production. **a** Growth curve of NZ-IL36γ cells. The growth of NZ-IL36γ cells was monitored by measuring the OD_600_ at each time point (0, 1, 3, 6, 12, 24, and 48 h). **b**,** c** Expression of rmIL-36γ at each time point. Western blotting was performed to analyze the rmIL-36γ levels in the cellular extract (**b**) and culture supernatant (**c**) samples. The arrowhead indicates the signal peptide-conjugated form of rmIL-36γ (27.8 kDa). The arrow indicates the signal peptide-cleaved form of rmIL-36γ (25.0 kDa). M: molecular mass markers (kDa). The protein expression levels quantified by ImageJ are shown below the blots. The peak expression time points are shown in green. Three independent experiments were performed. Values represent the means ± SD

The optimal culture time for maximizing the efficiency of rmIL-36γ production was determined. NZ-IL36γ cells were incubated for 0, 1, 3, 6, 12, 24, or 48 h after nisin induction. After measurement of the OD_600_, a cell extract was prepared for each time point, and rmIL-36γ expression was confirmed by western blotting using the anti-His tag Ab. The turbidity increased greatly after the addition of nisin, peaking after 6 h, suggesting that the exponential phase lasted until 6 h. Subsequently, the stationary phase began (Fig. 2a). In the western blotting analysis, the intensity of the bands corresponding to rmIL-36γ peaked at 6 h after nisin induction in the cellular extract samples (Fig. 2b), and in the supernatant samples, it peaked at 6 h to 24 h, and decreased after 48 h (Fig. 2c). During the peak from 6 to 24 h in the supernatant samples, the rmIL-36γ concentration was determined to be 349 ng/mL by ELISA.

### Viability of orally administered NZ-IL36γ in mouse intestines

**Fig. 3:**
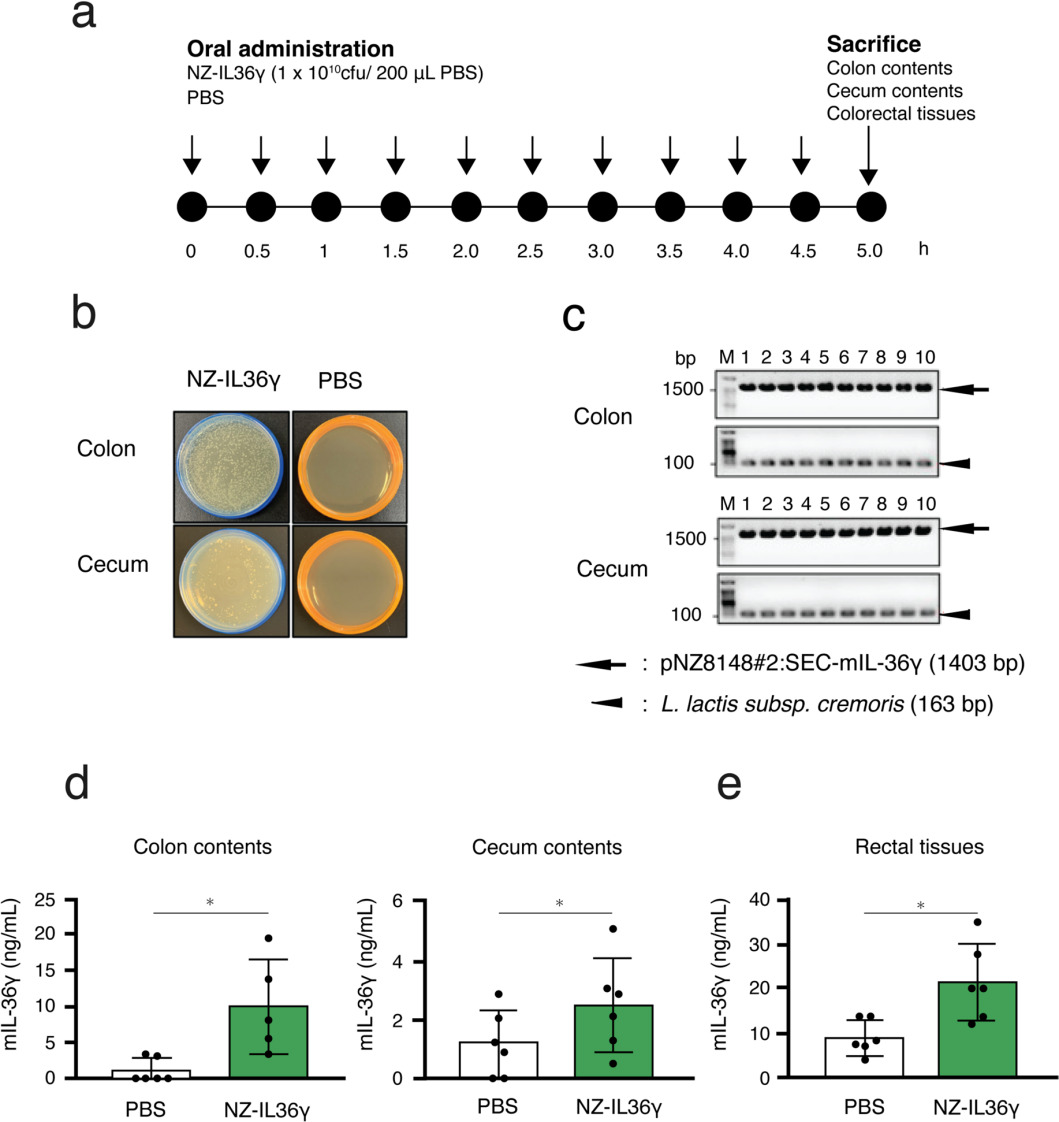
Viability of NZ-IL36γ cells and the levels of secreted ILs in mouse intestines. **a** The oral administration schedule. After 1 week of acclimatization, 12 mice were randomly assigned to two groups: one group (*n* = 6) was administered 1 × 10^10^ cfu/200 µL of PBS of NZ-IL36γ, and the other group (*n* = 6) was administered PBS via oral gavage every 30 min for a total of 10 times in a day. After all mice were sacrificed, the colon contents, cecum contents, and rectal tissues were collected from each mouse. **b** The ruminal contents from the colon (upper row) and cecum (bottom row) were cultivated on GM17cm agar. NZ-IL36γ group: left column, PBS group: right column. Representative photos are shown. **c** Agarose gel electrophoresis of the colony PCR products. Ten colonies were selected randomly from each agar plate, and amplification was performed using two primer pairs specific for pNZ8148#2:SEC-mIL-36γ (upper, arrow, 1403 bp) and *L. lactis* subsp. *cremoris* (bottom, arrowhead, 163 bp). Each experiment was performed at least three times. **d**,** e** The mIL-36γ concentrations of the colon contents (**d**; NZ-IL36γ: *n* = 5; PBS: *n* = 6), cecum contents (**d**; NZ-IL36γ: *n* = 6; PBS: *n* = 6), and rectal tissues (**e**; NZ-IL36γ: *n* = 6; PBS: *n* = 6) were quantified by ELISA. *p < 0.05 was considered to be statistically significant

To examine whether orally administered NZ-IL36γ cells are viable in mouse intestines, the homogenized ruminal contents were incubated on GM17cm agar, and colony PCR was performed using NZ-IL36γ-specific primer pairs. The oral administration schedule is shown in Fig. 3a. The mIL-36γ concentrations of the ruminal contents and rectal tissues were quantified by ELISA. In the NZ-IL36γ group, colonies were detected from both the colon and cecum contents (Fig. 3b). In contrast, no colonies were detected in the PBS group (Fig. 3b). Colony PCR products corresponding to the expected sizes were detected for each of the primer pairs (Fig. 3c; pNZ8148#2:SEC-mIL-36γ: 1,403 bp; *L. lactis* subsp. *cremoris*: 163 bp).

### Secretion of rmIL-36γ from NZ-IL36γ in mouse intestines

To evaluate how the NZ-IL36γ cells affect the mIL-36γ concentration in mouse intestines, the mIL-36γ concentrations in the colon, cecum, and rectal tissues were determined using ELISA. The mIL-36γ concentration of the colon contents from the NZ-IL36γ group (*n* = 5) was 9.99 ± 2.94 ng/mL, which was significantly higher than that of the PBS group (*n* = 6; 1.06 ± 0.67 ng/mL; Fig. 3d). The mIL-36γ concentration of the cecum contents from the NZ-IL36γ group (*n* = 6) was 2.50 ± 0.66 ng/mL, which was significantly higher than that of the PBS group (*n* = 6; 1.19 ± 0.47 ng/mL; Fig. 3d). Moreover, the mIL-36γ concentration of the rectal tissues was 21.31 ± 3.55 ng/mL, which was significantly higher than that of the PBS group (*n* = 6; 8.95 ± 1.60 ng/mL; Fig. 3e).

### Changes in the intestinal microbiome due to NZ-IL36γ administration

**Fig. 4:**
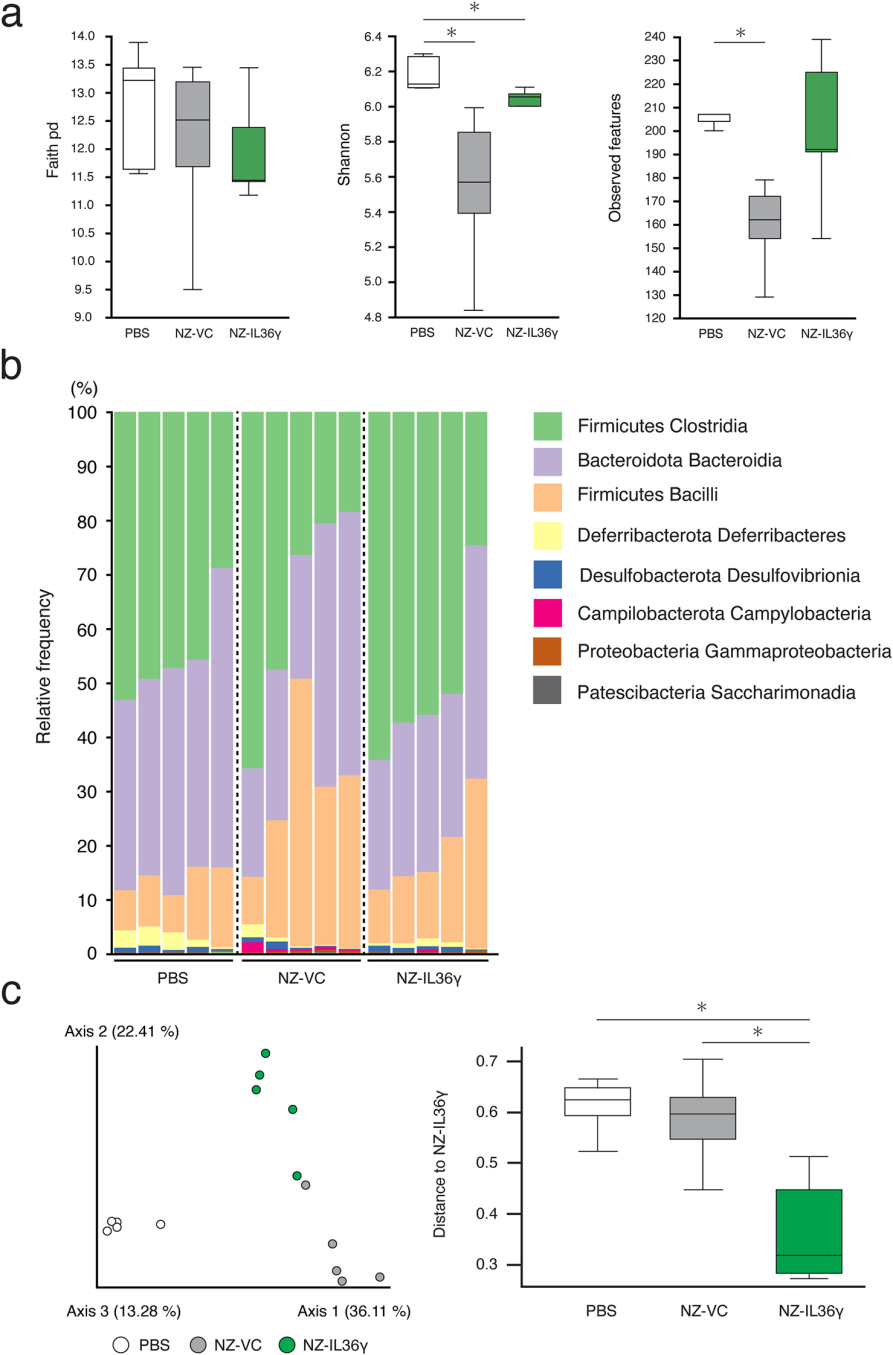

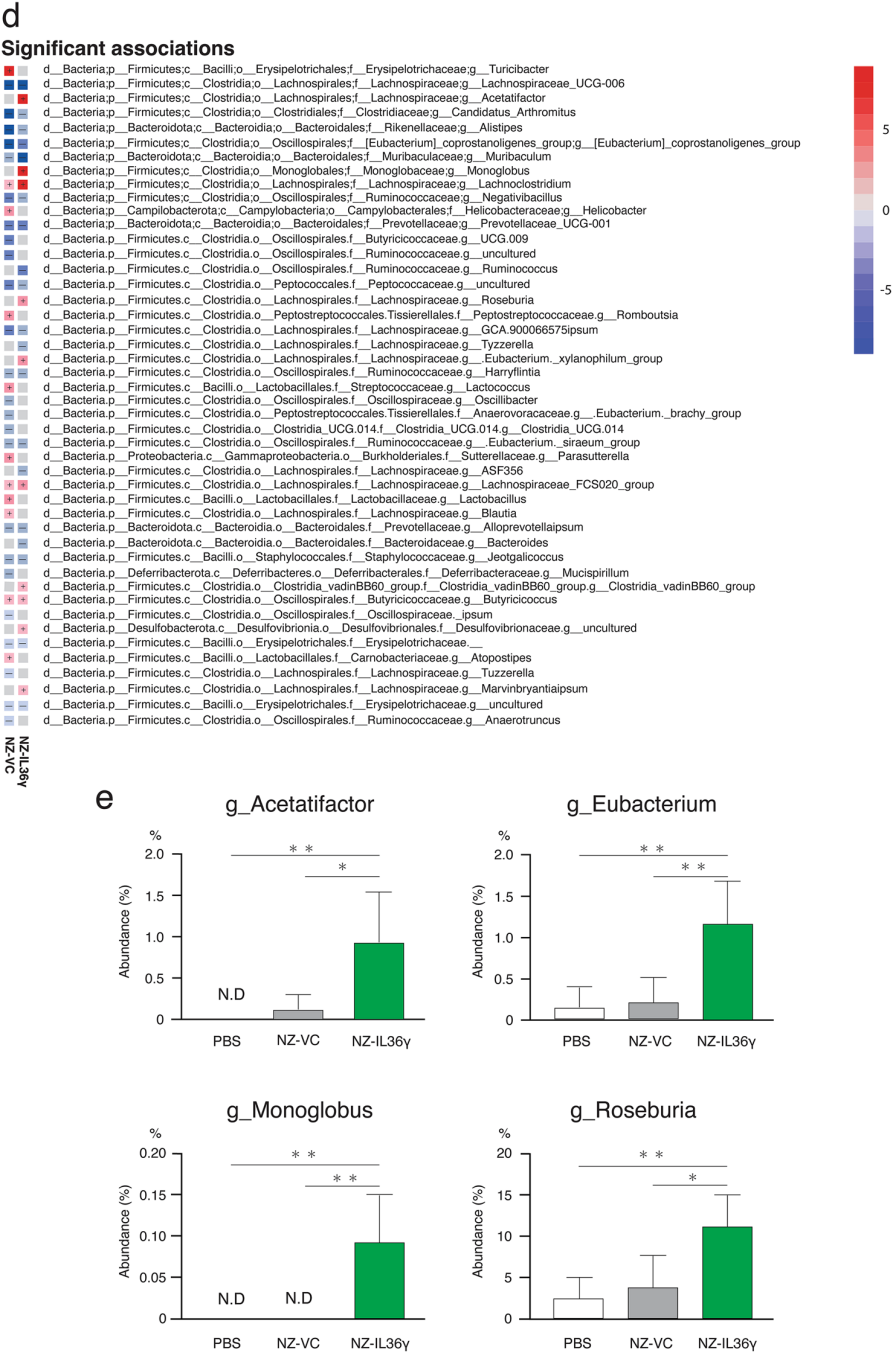
Changes in the gut microbiota due to NZ-IL36γ administration. Mice were treated with PBS (*n* = 5), NZ-VC (*n* = 5), or NZ-IL36γ (*n* = 5). Fecal samples were collected at the end of the administration period (day 14). **a** Boxplots depicting the α-diversity indices of the microbiota composition in mice. **b** The bacterial composition (%) at the class level is shown as a bar plot. **c** Left: The β-diversity of the intestinal microbiota composition in mice displayed by the Bray-Curtis distances. Right: Distances of the microbiota composition between NZ-IL36γ and PBS, and between NZ-IL36γ and NZ-VC. *p < 0.05 was considered to be statistically significant. **d** Genera association with population characteristics. The correlation and statistical significance between PBS and NZ-VC, and between PBS and NZ-IL36γ were determined by MaAsLin2 with multiple comparison adjustment by the false discovery rate. Taxa that are depicted were filtered for an FDR-adjusted *p* < 0.25 in comparison to the PBS group. An FDR-adjusted *p* < 0.25 was considered to be statistically significant. **e** Taxa in the NZ-IL36γ group that showed a significant increase with a *p* < 0.05 when compared to the PBS and NZ-VC groups are shown. *p < 0.05, **p < 0.01

We investigated whether NZ-IL36γ administration resulted in any alterations in the composition of the intestinal microbiota by 16 S V3-V4 rRNA gene sequencing (DDBJ BioProject accession number: PRJDB18230). Compared to the PBS group, the Shannon diversity index, a measure of the diversity of the gut microbiome, was significantly lower in the NZ-VC and NZ-IL36γ groups (*p* < 0.05), and the observed features were significantly lower in the NZ-VC group (*p* < 0.05), but not in the NZ-IL36γ group (Fig. 4a). No significant difference was found in Faith’s PD (Fig. 4a). To compare the differences in the bacterial composition between the PBS, NZ-VC, and NZ-IL36γ groups, a taxonomic analysis was performed using QIIME2 with the bacteria in each group plotted in a bar plot (Fig. 4b) The diversity between samples was further assessed by β-diversity analysis. The principal coordinate plot revealed a separation of the gut microbiota composition (Fig. 4c left), and the distances of the gut microbiota composition were significantly different between the NZ-IL36γ and PBS groups (*p* < 0.05), and between the NZ-IL36γ and NZ-VC groups (*p* < 0.05; Fig. 4c, right). We next analyzed the gut microbiota at the genus level using MaAsLin2. We were able to identify significant differences in the abundances of taxa between the PBS and NZ-VC groups, and between the PBS and NZ-IL36γ groups (Fig. 4d). The genera *Acetatifactor*, *Eubacterium*, *Monoglobus*, and *Roseburia* were more abundant in the NZ-IL36γ group than in the PBS and NZ-VC groups (Fig. 4e).

#### NZ-IL36γ enhances intestinal *Muc2 *expression in Mice.

**Fig. 5:**
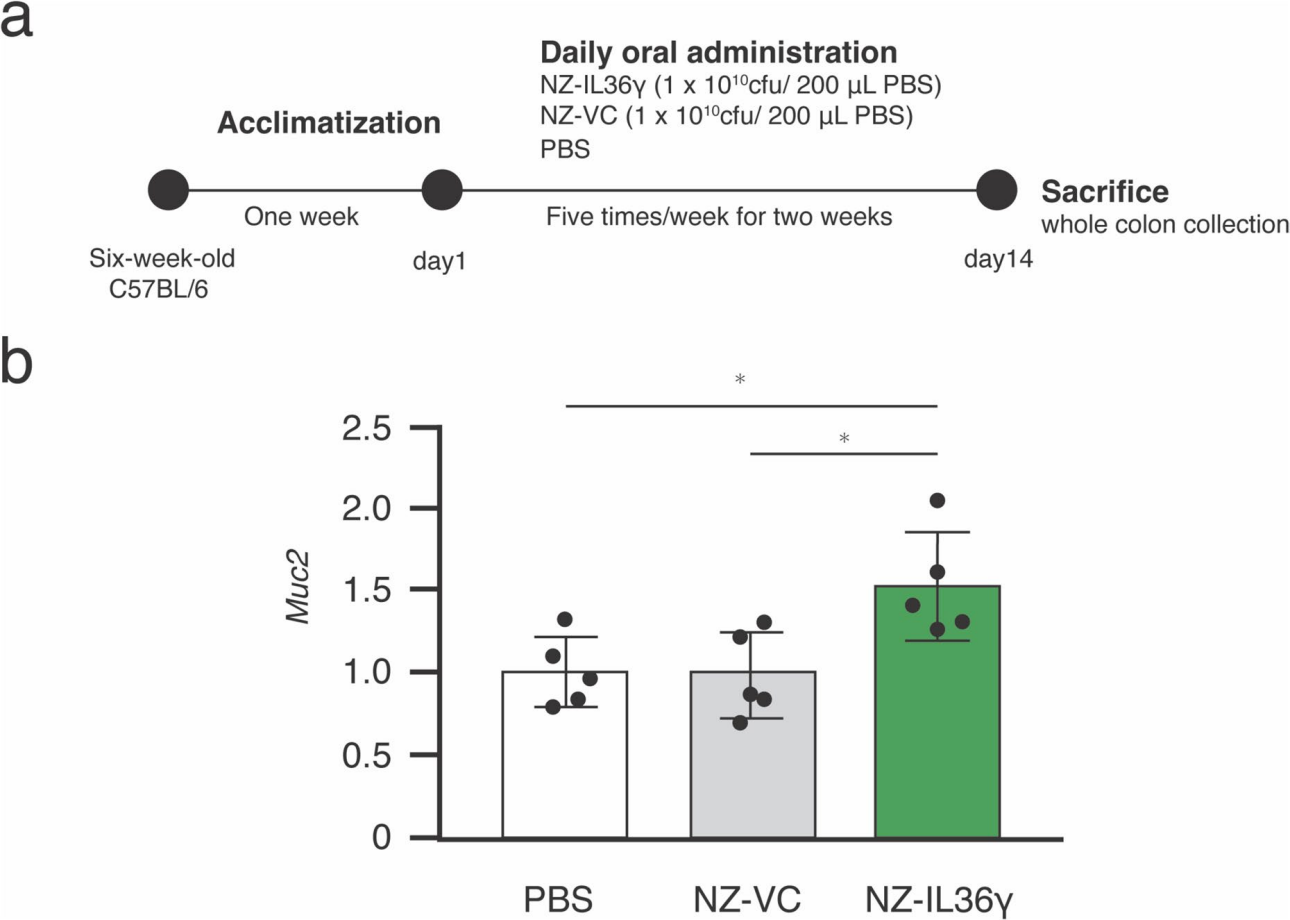
NZ-IL36γ administration promoted *Muc2* expression in the intestines of C57BL/6 mice. **a** The oral administration procedure. Six-week-old mice were acclimatized for 1 week, then assigned to three groups: one group (*n* = 5) was administered PBS, and the others were administered NZ-IL36γ (*n* = 5) or NZ-VC (*n* = 5) at 1 × 10^10^ cfu/200 µL of PBS via oral gavage five times per week for 2 weeks. At the end of the administration period (day 14), the mice were sacrificed, and the whole colons were collected for gene expression analyses. **b** RT-qPCR results for the *Muc2* expression levels in the whole colon of mice. Data are expressed as the relative values versus those of the PBS controls. *p < 0.05 was considered to be statistically significant

We also evaluated the effects of NZ-IL36γ administration on the level of *Muc2* expression in the intestine. We measured the mRNA expression level by RT-qPCR. After 1 week of acclimatization, 7-week-old mice were administered PBS (*n* = 5), NZ-VC (*n* = 5), or NZ-IL36γ (*n* = 5) five times per week for 2 weeks (Fig. 5a). RT-qPCR was performed with the whole colon, and data are expressed as the relative values versus those of the PBS group. The *Muc2* levels were significantly higher in the NZ-IL36γ group than in the NZ-VC group (*p* < 0.05; Fig. 5b).

#### Production of rmIL-36γ by NZ-IL36γ promotes *Muc2* expression in CMT93/69 and HT-29 cells

**Fig. 6:**
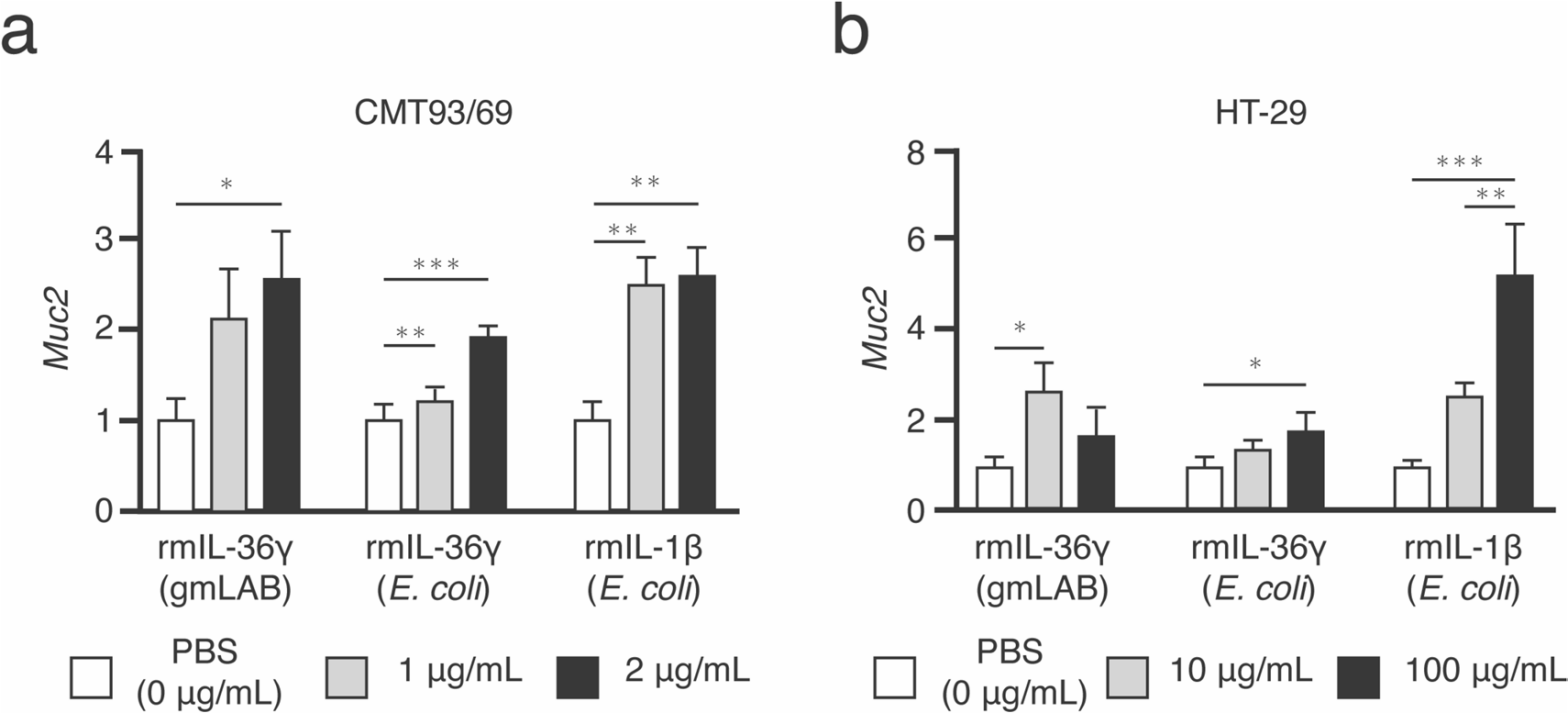
RT-qPCR results for the bioactivity of rmIL-36γ in promoting *Muc2* expression in CMT93/69 and HT-29 cells. CMT93/69 (**a**) and HT-29 (**b**) cells were stimulated with various concentrations of purified rmIL-36γ (from gmLAB), rmIL-36γ (from *E. coli*), or rmIL-1β (from *E. coli*) for 24 h. Data are expressed as relative values versus those of the PBS control (0 µg/mL of rmIL-36γ) or as the means ± SD of three independent experiments. *p < 0.05, **p < 0.01, and ***p < 0.001 were considered to be statistically significant

The ability of rmIL-36γ to promote *Muc2* expression was investigated in CMT93/69 murine colorectal epithelial cells and HT-29 human colonic cells using various concentrations of purified rmIL-36γ (from gmLAB), rmIL-36γ (from *E. coli*; positive control), and rmIL-1β (from *E. coli*; positive control). In the CMT93/69 cells, *Muc2* expression gradually increased in a rmIL-36γ (from gmLAB) concentration-dependent manner, and was significantly stimulated by 2 µg/mL of rmIL-36γ (from gmLAB) when compared to PBS (0 µg/mL of rmIL-36γ; *p* < 0.05; Fig. 6a). Similarly, *Muc2* expression was stimulated by both of the positive controls (rmIL-36γ (from *E. coli*) and rmIL-1β (from *E. coli*)), i.e.,* Muc2* expression gradually increased in a concentration-dependent manner (Fig. 6a). In contrast, in the HT-29 cells, a significant increase in *Muc2* expression was induced by 10 µg/mL of rmIL-36γ (from gmLAB; *p* < 0.05; Fig. 6b), while 100 µg/mL of rmIL-36γ (from gmLAB) did not significantly increase the expression level when compared to PBS (0 µg/mL of rmIL-36γ). Both of the positive controls (rmIL-36γ (from *E. coli*) and rmIL-1β (from *E. coli*)) gradually increased *Muc2* expression in a concentration-dependent manner (Fig. 6b).

## Discussion

Several studies have provided strong evidence for a link between IL-36 and mucosal immunity (Kovach et al. 2017; Giannoudaki et al. 2019). For example, IL-36 cytokines have been reported to enhance colonic mucus secretion in mice lacking IL-36Ra, an IL-36 family signaling antagonist, which suggested that IL-36 cytokines lead to increased mucus production in the colon (Giannoudaki et al. 2019). Another study has indicated that IL-36γ is induced and released from cells as a result of pathogenic damage, and is subsequently activated as a direct result of proteolytic virulence factors secreted by invasive epithelial pathogens (Macleod et al. 2020). This suggests that IL-36γ functions as a global epithelial alarmin and broad sensor of pathogenic infection. In addition, induced IL-36γ influences neutrophil accumulation, cytokine production, and the repair of intestinal damage following injury through the IL-36γ/IL-36 receptor signaling pathway (Medina-Contreras et al. 2016; Ngo et al. 2018). These studies showed that IL-36γ is a key initiator of immune responses, and that it has an intestine-protecting role. Thus, we focused on IL-36γ. We established a gmLAB that synthesizes and secretes rmIL-36γ, and we studied how rmIL-36γ affects the intestinal microbiome and colonic mucus gene expression.

NICE in *L. lactis* is among the most common and efficient ways to obtain a desired recombinant protein, and to develop a mucosal delivery system for proteins using gmLAB (Mierau and Kleerebezem 2005). In the present study, we constructed a NICE-based secretion system for rmIL-36γ in NZ9000 cells using a secretion vector (pNZ8148#2:SEC-mIL-36γ) with an expression cassette for a fusion peptide comprising a lactococcal signal peptide (27 amino acids, DNA sequence location 204 to 284 in pNZ8148#2:SEC-mIL-36γ) (Shigemori et al. 2014), His-tag, and mIL-36γ under the control of the nisin A promoter. Western blotting results showed that following nisin stimulation, pNZ8148#2:SEC-mIL-36γ-transformed NZ9000 (NZ-IL36γ) cells produced an intracellular His-tagged protein corresponding in size to the signal peptide-conjugated form of rmIL-36γ (27.8 kDa). The results also indicated that the produced proteins were secreted using the secretion machinery of the cells, given that the western blotting band observed from the culture supernatant corresponded to the signal peptide-cleaved form of rmIL-36γ (25.0 kDa).

The growth curve for NZ-IL36γ showed that the exponential phase began after the addition of nisin and continued for 6 h, peaking at 6 h. The expression level in the cellular extract samples was highest at 6 h, while that in the supernatant samples peaked at 6 to 24 h. During the peak from 6 to 24 h in the supernatant samples, the rmIL-36γ concentration of the supernatant was 349 ng/mL. Several studies have reported the recombinant IL concentrations from the NICE system, e.g., the concentrations were 2000 ng/mL for mouse IL-1Ra (Namai et al. 2020a), 2 ng/mL for mouse IL-2 (Fernández et al. 2007), 580 ng/mL for porcine IL-2 (Avall-Jääskeläinen and Palva 2006), 40 ng/mL for mouse IL-10 (Bermúdez-Humarán et al. 2015), 0.185 ng/mL for mouse IL-12 (Fernandez et al. 2009), 0.7 ng/mL for mouse IL-18 (Feizollahzadeh et al. 2016), and 10 ng/mL for human IL-22 (Loera-Arias et al. 2014). These data suggest that the concentration differs for different types of recombinant proteins. In this study, we succeeded in constructing a gmLAB that hypersecretes rmIL-36γ.

The viability of lactic acid bacteria in the digestive tract has been studied for many different species and conditions  (Drouault et al. 1999, Kimoto et al. 2003, Namai et al. 2020). However, it was unknown whether NZ-IL36γ cells could survive in the digestive tract. Thus, the viability of NZ-IL36γ cells and the secretion of IL from these cells were studied in the digestive tract of mice to examine the fate of the ingested NZ-IL36γ after oral administration. For this purpose, we used selection ager (GM17cm) to differentiate the gmLAB from other intestinal bacteria. Two specific primer pairs for pNZ8148#2:SEC-mIL-36γ and *L. lactis* subsp. *cremoris* were used for colony PCR to specifically detect NZ-IL36γ. Colonies on GM17cm and specific PCR products were detected in both the colon and cecum contents. Moreover, the IL-36γ concentration was significantly higher in all samples (the colon contents, cecum contents, and rectal tissues) from the NZ-IL36γ group than those from the PBS group. These results indicated that NZ-IL36γ cells were viable and were secreting rmIL-36γ in the colon.

It has been reported that microbial communities are implicated in the maintenance of intestinal homeostasis. IL-36 cytokines have been previously shown to alter the composition of the intestinal microbiome in mice with an IL-36 receptor antagonist deficiency (Giannoudaki et al. 2019), suggesting that IL-36 cytokines are a crucial factor for microbial communities. Thus, we next investigated whether the administration of NZ-IL36γ cells resulted in any alterations in the components of the intestinal microbiome by 16 S rRNA gene sequencing. To evaluate the α-diversity, which shows the diversity of organisms in an ecological community, we analyzed several indices (Willis 2019). The Shannon diversity index was significantly lower in the NZ-VC and NZ-IL36γ groups than in the PBS group, and was slightly higher in the NZ-IL36γ group than in the NZ-VC group (Fig. 4a). The observed features index was also significantly lower in the NZ-VC group than in the PBS group, with no significant difference between the PBS and NZ-IL36γ groups. These data suggested that while NZ-VC decreased the α-diversity, the administration of NZ-IL36γ, which secretes rmIL-36γ, helped maintain the α-diversity in the gut of the mice.

Notably, a relative increase in the abundances of *Acetatifactor*, *Eubacterium*, *Monoglobus*, and *Roseburia* species was found to be specifically dependent on NZ-IL36γ administration in the mice. Recently, results from a comparative analysis between IL-1β-knockout and wild-type mice have suggested that IL-1β induces the mRNA expression of angiogenin 4, which has a potential antimicrobial effect, and causes a change in the intestinal microbiota (Bechberger et al. 2023). Similarly, in the present study, it was shown that the administration of NZ-IL36γ, which secretes IL-36γ belonging to the IL-1 superfamily, led to changes in the intestinal microbiota of mice, although direct evidence is pending. Interestingly, *Acetatifactor*, which was recently identified as the bacterial genus most closely related to *Clostridium cluster XIV* (Pfeiffer et al. 2012), and *Eubacterium* species have been reported to possess 7α-dehydroxylase enzymes that convert primary bile acids, cholic acid and chenodeoxycholic acid into deoxycholic acid (Mukherjee et al. 2020). A previous report has shown that bile acids (specifically deoxycholic acid) induced *Muc2* overexpression in human esophageal adenocarcinoma cells through the activation of NF-κB transcription (Wu et al. 2008). These data suggest that the administration of NZ-IL36γ cells might have promoted the growth of *Acetatifactor* and *Eubacterium* species, and consequently induced *Muc2* overexpression. In addition, *Monoglobus* species are enriched in healthy individuals (Chen et al. 2022), and they have the ability to ferment dietary fiber to produce butyrate (Parada Venegas et al. 2019), which inhibits inflammation and oxidative stress (Hu et al. 2013). Also of note is that *Roseburia* species accounted for more than 10% of the total bacteria in the NZ-IL36γ group. Recently, a link has been reported between *Roseburia* species and gut health, including chronic inflammatory diseases, such as UC and CD. It has been reported that patients with UC had lower counts of *Roseburia* in their fecal samples (Vermeiren et al. 2012; Machiels et al. 2017). Similarly, the disappearance or a significant decrease of *Roseburia* species has been reported as a specific change seen in the intestinal microbiota of patients with ileal CD (Willing et al. 2010; Chen et al. 2014). Thus, in light of the gut microbiota alterations and increased abundances of the genera *Acetatifactor*, *Eubacterium*, *Monoglobus*, and *Roseburia* observed in the present study, it appears that the constructed NZ-IL36γ not only promotes *Muc2* expression, but might also be associated with preventing intestinal diseases.

Although we showed that the NZ-IL36γ cells were viable, secreted rmIL-36γ, and induced changes in the intestinal microbiome, we needed to confirm whether they promoted *Muc2* expression. Thus, we conducted an in vivo mouse study in which the whole colon was collected after 2 weeks of oral administration, and RT-qPCR was performed. The relative *Muc2* expression level was significantly higher in the NZ-IL36γ group than in the NZ-VC and PBS groups. It has been reported that IL-36 cytokines interact with their specific heterodimeric receptor, which is composed of IL-36R and IL-1RAcP (Bassoy et al. 2018). Through IL-1RAcP binding, IL-36γ activates the pathway leading to NF-κB activation (Abdel-Latif et al. 2004; Towne et al. 2004; Li et al. 2006, 2007). These findings suggest that the increased *Muc2* expression level might be related to IL-1RAcP binding as a pathway separate from the bile acid pathway. Thus, we next investigated whether the mIL-36γ purified from NZ-IL36γ cells would induce *Muc2* expression in CMT93/69 cells.

The in vitro bioactivity of rmIL-36γ in inducing *Muc2* gene expression was examined by stimulating CMT93/69 cells with various concentrations of purified rmIL-36γ (from gmLAB), rmIL-36γ (from *E. coli*), and IL-1β (from *E. coli*). As colon crypt mucin, which is mainly encoded by *Muc2* (Johansson et al. 2008), is upregulated by cytokines, such as IL-1β (Kim et al. 2000), we used IL-1β as a positive control for inducing *Muc2* gene expression. We found that *Muc2* expression gradually increased in a rmIL-36γ (from gmLAB) concentration-dependent manner. *Muc2* expression was significantly stimulated by 2 µg/mL of rmIL-36γ (from gmLAB), suggesting that rmIL-36γ (from gmLAB) can promote mucus production in the mouse intestine. Similarly, an increase in mRNA expression was observed with rmIL-36γ (from *E. coli*), indicating that purified rmIL-36γ from both gmLAB and *E. coli* have a similar effect on *Muc2* expression. In terms of *Muc2* expression, the results of our in vivo and in vitro studies suggested that the secreted IL-36γ promotes the growth of *Acetatifactor* and *Eubacterium* species, and induces an intracellular signaling cascade; both of these lead to the activation of NF-κB, and thereby mediate *Muc2* expression. Although we demonstrated the effects of NZ-IL36γ cells in mice, we did not know whether the same would occur in humans. Thus, we analyzed the cross-reactivity of rmIL-36γ secreted by NZ-IL36γ to human colonic cell lines (Fig. 6b). The ability of rmIL-36γ to promote *Muc2* expression in HT-29 was investigated using recombinant mouse ILs. The rmIL-36γ (from gmLAB) at 10 µg/mL significantly increased *Muc2* expression when compared to the PBS control, and at 100 µg/mL, no significant increase was seen. In contrast, the positive controls (rmIL-36γ (from *E. coli*) and rmIL-1β (from *E. coli*) increased *Muc2* expression in an IL concentration-dependent manner, suggesting that mouse ILs have the same *Muc2* expression-promoting effects in CMT93/69. Thus, these data indicate that NZ-IL36γ might have similar effects in humans and mice, and might help improve the intestinal environment.

Although we demonstrated protective effects of NZ-IL36γ on the intestinal environment in mice, we did not examine the effects in pathological models. Therefore, further investigations on the effects of NZ-IL36γ are warranted in pathogenic models, such as in mice with intestinal bowel disease (Ordás et al. 2012; Namai et al. 2020a; Ma et al. 2022), and in IL-36γ-knockout mice.

In conclusion, we successfully developed a genetically modified strain of *L. lactis*, NZ-IL36γ, which hypersecreted rmIL-36γ under optimized culture conditions. We showed the viability of NZ-IL36γ cells in the mouse intestine, and the secretion of the IL from these cells. Moreover, we demonstrated that NZ-IL36γ increased the abundances of *Acetatifactor*, *Eubacterium*, *Monoglobus*, and *Roseburia* species in the mouse intestine, and that they were associated with *Muc2* expression and possibly with the prevention of intestinal diseases. In the subsequent in vitro experiment, the purified rmIL-36γ promoted *Muc2* expression in a bioactivity assay using murine colorectal epithelial cells. Finally, using a bioactivity assay with human colonic cell lines, we showed that NZ-IL36γ may have similar effects in humans. Taken together, our results suggest that NZ-IL36γ may be useful for improving the intestinal environment in humans.

## Data Availability

Sequence data of the resulting plasmid, pNZ8148#2:SEC-mIL-36γ, have been deposited in DNA data bank of Japan with accession number LC823121. Microbiota analysis data have been deposited in DNA data bank of Japan with accession number PRJDB18230.
